# Psychological and behavioral responses to the declaration of COVID-19 as a pandemic: A comparative study of Hong Kong, Singapore, and the U.S.

**DOI:** 10.1371/journal.pone.0275854

**Published:** 2022-10-10

**Authors:** Jingshi (Joyce) Liu, Anirban Mukhopadhyay, Catherine Wing-Man Yeung

**Affiliations:** 1 Faculty of Management, Bayes Business School (Formerly Cass), City, University of London, London, United Kingdom; 2 Department of Marketing, School of Business and Management, Hong Kong University of Science and Technology, Hong Kong, Hong Kong; 3 Department of Marketing, CUHK Business School, Chinese University of Hong Kong, Hong Kong; University of Hong Kong, HONG KONG

## Abstract

What is the effect of declaring a pandemic? This research assesses behavioral and psychological responses to the WHO declaration of the COVID-19 pandemic, in Hong Kong, Singapore, and the U.S. We surveyed 3,032 members of the general public in these three regions about the preventative actions they were taking and their worries related to COVID-19. The WHO announcement on March 11^th^, 2020 created a quasi-experimental test of responses immediately before versus after the announcement. The declaration of the pandemic increased worries about the capacity of the local healthcare system in each region, as well as the proportion of people engaging in preventative actions, including actions not recommended by medical professionals. The number of actions taken correlates positively with anxiety and worries. Declaring the COVID-19 crisis as a pandemic had tangible effects–positive (increased community engagement) and negative (increased generalized anxiety)–which manifested differently across regions in line with expectancy disconfirmation theory.

“*Pandemic is not a word to use lightly or carelessly*. *It is a word that*, *if misused*, *can cause unreasonable fear*, *or unjustified acceptance that the fight is over*, *leading to unnecessary suffering and death*.*”*

Dr. Tedros Ghebreyesus, Director-General, World Health Organization (WHO)

## Introduction

Over two years have passed since the outbreak of the COVID-19. There has been much speculation and finger-pointing about its origin, but evidence-based discussions of why the outbreak escalated into a catastrophic pandemic, and how the world could prepare for future pandemic threats, have only just begun. Having looked closely into the world’s early responses to the outbreak, the Independent Panel for Pandemic Preparedness & Response [1] identified two critical factors contributing to the escalation of the outbreak: An outdated alert system that was too slow for a fast-moving pathogen, and the wait-and-see approach adopted by many countries in response to the COVID-19 Public Health Emergency of International Concern (PHEIC) Declaration issued on January 30, 2020 by the WHO. According to the WHO, the PHEIC was the “loudest possible” alarm they could sound to signal that a pandemic might be imminent, to call for strong measures to detect the disease, isolate and treat cases, trace contacts, and promote social distancing measures. However, many parts of the world hesitated to take a full-scale response to the declaration. The Panel concluded that the delay, hesitation, and denial resulted in “a lost month” of opportunity in February 2020 to contain the outbreak.

In the month that followed the PHEIC declaration, the outbreak spread to more than thirty countries in nearly all continents. On March 11, 2020, the WHO declared the COVID-19 outbreak to be a worldwide pandemic. In the subsequent days, many countries started taking full-scale responses. There were strong debates within the medical community with some arguing that the WHO could have made the pandemic declaration earlier to signal its urgency [2]. Dr. Joanne Liu, one of the panelists of the Independent Panel, admitted that “the term PHEIC isn’t as sexy as an emotive word, such as ‘pandemic’ or ‘emergency’. But researchers and health officials chose it partly because they wanted to avoid panic while encouraging world leaders to act according to WHO advice to contain a threat.” Likewise, Dr. Tedros Ghebreyesus, Director-General of the WHO, explained the difficulties involved in labeling the outbreak a pandemic: “Pandemic is not a word to use lightly or carelessly. It is a word that, if misused, can cause unreasonable fear, or unjustified acceptance that the fight is over, leading to unnecessary suffering and death.” Declaring a pandemic is clearly an important policy decision, and the WHO’s decision revealed a difficult trade-off [3].

Why was this decision so difficult? The above quote reveals two fundamental questions that underlie the struggle in using the P-word. First, it is possible that the declaration itself may incite unreasonable fear in the general public. As the Director of the WHO’s Collaborating Center on National and Global Health Law, Lawrence Gostin, observed, “the word ‘panic’ is literally in the word ‘pandemic’” [4]. And second, it is not known whether the declaration would induce people to step up their preventative behaviors, or might they instead become paralyzed by the fear? While it is hard to predict, ex-ante, the exact psychological and behavioral responses of the public, psychological research can inform such predictions. The health promotion literature posits that hard-hitting (fear-inducing) messages may spur people to increase preventative behaviors, but might also trigger panic responses that cause counterproductive reactions [5, 6]. The label “pandemic,” which indicates severe infection and explosive transmissibility of a disease, could indeed unleash fear that there is zero immunity in the population. This reasoning appears closely aligned with the WHO’s worries.

Fear is a double-edge sword in public health messaging [6]. Fear induced by hard-hitting public health messages can motivate people to address the fear-inducing threat by changing their behaviors [5–9]. For example, fear of lung cancer may motivate people to quit smoking, and fear of AIDS may motivate people to practice safe sex. However, fear may also trigger defensive responses such as denial of the problem or its existence (e.g., “drinking is not the only cause of traffic accidents”, [10]), perceived personal invulnerability (e.g., “It will not affect me because I’m young”) and avoidance of preventative actions [5, 8]. If not properly addressed, fear can cause anxiety and panic, adversely affecting mental health and spurring irrational behaviors [11, 12]. Essentially, fear-inducing messages backfire if the fear experienced makes people feel they are losing control over the situation [13], or if the fear generalizes to other domains of life causing anxiety or panic [14, 15]. As such, even though the fear of COVID-19 has been shown to positively correlate with compliance with public health measures [16], public health experts cautioned about the use of fear appeals in health communications pertaining to COVID-19 [17].

The declaration of COVID-19 as a pandemic might potentially have had similar effects. A pandemic is a disease that extends over large geographic areas and exhibits a high attack rate with explosive spread due to high transmissibility and low immunity [18]. Prior to the COVID-19 pandemic declaration, the media had been reminding the public about other deadly pandemics in history. As a result, the public might have developed fear and anxiety towards the categorical label “pandemic,” regardless of other specific considerations such as the potential fatality and severity of COVID-19 in particular [19].

So, under what conditions can a pandemic declaration lead to positive outcomes among the general public, such as increasing community engagement in preventative actions? Meta-analysis [20] shows that fear communications are most effective when people know what specific actions to take to address the fear-inducing threats. That is, when people know the actions they can take to address the threat, and are able to implement these actions, fear can promote desirable behavioral change, without necessarily experiencing anxiety or panic [14, 15]. In the case of COVID-19, in Spring 2020, medical professionals had provided clear and practical guidance regarding preventive measures: frequent hand washing, covering coughs and sneezes, not touching one’s mouth, eyes, or nose, and practicing social distancing (wearing masks had not been recommended by the WHO at first). Thus, people may respond to the pandemic declaration by acting in these recommended ways. Nevertheless, the potential positive effects of these recommendations may be undermined by misinformation and fake cures spread via social media [21–23]. If people are misinformed about preventative measures, not only may they not take the right actions, they may also suffer in terms of psychological outcomes.

Scientists have warned that COVID-19 will not be the last pandemic [24]. Thus, it is important to look into the empirical evidence to understand how people respond to the declaration of the pandemic and related policies. Emerging research has investigated people’s psychological responses to COVID-19 [25], as well as their attitudes toward specific preventative measures and treatments of the disease [26, 27]. Building on the prior research, here, we focus on understanding people’s reactions to the declaration of the pandemic, in particular, how people’s psychological and behavioral responses toward COVID-19 *change* as a function of the declaration.

## Method

### Overview

In this research, we assessed people’s psychological and behavioral responses right before and right after the pandemic declaration. We conducted a pre-registered survey with human participants’ approval (described below; link to pre-registration: https://osf.io/ckg25/?view_only=5de605995c0047e2a019cee931186534). The sample size was pre-determined to be 3,000 before data collection, and our final sample size was 3,032 (see S1 Appendix in S1 File). Data were collected in the U.S., Hong Kong, and Singapore, from March 8^th^, 2020 to March 18^th^, 2020. These three regions were chosen because they represented different stages of the virus outbreak and different levels of trust in the WHO. Specifically, in early March, the COVID-19 outbreak had already started for over a month in Hong Kong and Singapore, but had just begun in the U.S. Compared to those in Hong Kong and Singapore, the U.S. citizens had a lower expectancy of outbreak. Further, media reports suggested that anxiety was lower in Singapore than in Hong Kong. In Hong Kong, schools were closed and work-from-home policies started from late January; such policies were not put in place until April in Singapore and the U.S. Therefore, respondents from the three regions varied in terms of their levels of expectancy and anxiety before the pandemic declaration, and were taking different preventative actions. Thus, data from these three places should capture meaningful variations in experiences with and expectations about COVID-19. As discussed, potential differences in psychological and behavioral responses to the pandemic announcement across these regions can have implications for healthcare policy announcements.

We measured self-reported practice of the WHO-recommended behaviors before versus after the pandemic declaration. We also measured people’s worries about whether they would get appropriate treatment if they contracted the disease, and whether the local healthcare system would be able to accommodate them. These were genuine threats the COVID-19 outbreak carried for individuals and societies, and, as the last two years have sadly borne out, fear about these outcomes was justifiable. However, fear that spilled beyond these considerations was less “reasonable” and could have lasting effects [28, 29]. We assessed this using a measure of generalized anxiety to gauge fear and anxiety that were generalized to other aspects of life. In addition, we included self-reported behavioral measures of two fake cures (keeping one’s throat moist, doing a deep breathing test, [22]) to gauge whether respondents were unreasonably fearful, as anxiety might increase susceptibility to false information (such as myths about preventative measures circulated on social media, [30]; cf. [31]). Finally, we asked respondents to indicate which of several product categories (e.g., toilet paper) they had stockpiled. An increase in stockpiling might reflect people’s perceptions of the threat to their day-to-day life.

### Human participants

Our research design and procedure were reviewed and approved by the Human Participants Research Panel at the Hong Kong University of Science and Technology (reference number: HPR #464), the institution with which the first and second authors were affiliated at the time of conducting this research. Because our research involved data collection in multiple countries, we employed Qualtrics Panels, an international survey research agency that offers researchers access to samples with specified demographic characteristics around the world. Respondents were recruited by Qualtrics Panels following their standard informed consent procedure. All respondents supplied written informed consent to participate in surveys sent to them by Qualtrics Panels, including the current survey. The consent practices were in accordance with the local data collection regulations. All respondents voluntarily completed the study online for monetary compensation.

Our intention, as pre-registered, was to recruit adult participants only. However, Qualtrics Panels recruited some respondents under the age of 18 (accounting for 1.6% of the final sample), on the assurance that these respondents had permission from their guardian, and the parent/guardian might have done the survey for them. We followed our pre-registered protocol and retained these participants for analysis. Data were analyzed anonymously.

### Procedure

The first part of the survey measured respondents’ knowledge about the WHO’s recommended preventative actions regarding COVID-19. Respondents were asked to identify the four recommended actions (hand washing, social distancing, practicing respiratory hygiene, and not touching eyes, hands, or mouth) out of a list of eight given actions. Four other actions (see Table 1) were sourced from social media. Respondents indicated which of these eight actions they personally were taking. We then indicated to respondents the four actions that were recommended by the WHO, or the ones sourced from social media. (While this was an initial focus of our study, and several of our predictions as pre-registered were supported by the data, we do not focus on this here; see S2 Appendix in S1 File for more detail). Respondents reported their generalized anxiety [32], and various concerns, behaviors, and the extent to which they trusted different sources (S3 Appendix in S1 File). Finally, we collected information about demographics and pertinent medical conditions. The survey was conducted in English in the U.S. and Singapore, and in Chinese in Hong Kong. The first and third authors, who are native Chinese speakers, translated and back-translated the survey to Chinese for data collection in Hong Kong. All data are available at: https://osf.io/cpyh3/?view_only=33e403d5781a40beba86228c3146811b.

**Table 1:** Anxiety and worries before and after the WHO pandemic announcement, by region: Hong Kong, Singapore, and the U.S., 2020.

Variable	Hong Kong		Singapore		U.S.		Overall *F*-value (*p*)
	Before	After	Before	After	Before	After	
(*N* = 3,032)	763	257	697	308	834	173	
**Generalized Anxiety**							4.29 (0.01)
Mean	5.61	6.48	4.64	4.9	5.14	7.09	
*SD*	5.21	5.3	5.19	4.85	5.52	6.61	
95% CI for difference	(0.11, 1.63)		(−4.56, 0.97)		(1.07, 2.82)		
**Worry that the outbreak will exceed the healthcare system’s capacity**							4.18 (0.02)
Mean	5.16	5.41	3.36	3.69	3.85	4.62	
*SD*	1.54	1.45	1.61	1.69	1.91	1.96	
95% CI for difference	(0.02, 0.50)		(0.11, 0.56)		(0.49, 1.05)		
**Worry about not being able to receive treatment personally**							3.13 (0.04)
Mean	4.96	5.27	3.36	3.75	3.66	4.45	
*SD*	1.63	1.54	1.81	1.9	2.02	2.17	
95% CI for difference	(0.05, 0.57)		(0.14, 0.64)		(0.49, 1.09)		

SD = standard deviation; CI = confidence interval.

During our data collection, on March 11^th^, the WHO declared COVID-19 to be a global pandemic. This announcement created a quasi-experimental context because other external factors were relatively stable during the short period of our data collection. We thus examined how people’s psychological and behavioral responses varied as a function of the pandemic announcement, across the three geographic regions. We focus here on three psychological responses–generalized anxiety, concerns about the healthcare system capacity, and concerns about receiving treatment personally, and two behavioral responses–preventative actions taken and stockpiling. Results on the other variables are available upon request.

To explore changes in responses after the pandemic announcement, following Li and colleagues, 2020 [33], we compared data collected before and after the date of the announcement (March 11^th^), and conducted separate analyses using the critical date and geographic regions as factors. We analyzed respondents’ psychological and behavioral responses, first reporting the results across regions before the pandemic declaration, and then reporting the contrast within each region before versus after the announcement [34].

## Results

### Changes in psychological responses

#### Generalized anxiety

We computed the generalized anxiety score (GAD) by summing the scale items. The final score ranged from 0 to 21. We used “10” as the threshold to determine whether respondents were “moderately to severely anxious,” as this is the clinical threshold for screening of Generalized Anxiety Disorder [32]. Before the pandemic declaration, 19.4%, 20.2%, and 16.1% respondents in the U.S., Hong Kong, and Singapore, respectively, were moderately to severely anxious. After the declaration, however, the proportion significantly increased to 32.9% in the U.S. (χ^2^ = 15.40, *p* < 0.01). Such an increase was not observed in Hong Kong (24.1%, χ^2^ = 1.79, *p* = 0.18) or Singapore (17.5%, χ^2^ = 0.33, *p* = 0.58). Thus, a significantly greater proportion of respondents in the U.S., but not in Hong Kong or Singapore, experienced anxiety post-announcement.

An ANOVA on mean anxiety scores (Table 1) revealed that generalized anxiety varied as a function of the pandemic declaration, geographic region, and their interaction (*F*(2, 3026) = 4.29, *p* = 0.01). Before the declaration, the U.S. and Hong Kong had similar anxiety levels (*M*_US_ = 5.14, *M*_HK_ = 5.61; *p* = 0.08). In Singapore (*M* = 4.62), anxiety was significantly lower than Hong Kong (*p* < 0.001) and marginally lower than the U.S. (*p* = 0.07). After the declaration, generalized anxiety increased the most in the U.S. (*M* = 7.09, *p* < 0.01, *d* = 0.32), somewhat less in Hong Kong (*M* = 6.48, *p* = 0.03, *d* = 0.17), and stayed almost the same in Singapore (*M* = 4.90, *p* = 0.49, *d* = 0.05).

#### Worry about healthcare capacity

Respondents indicated the extent to which they worried about the outbreak exceeding the healthcare system’s capacity (1 = *not at all*; 7 = *very much*; Table 1). The level of worry varied as a function of the declaration, region, and their interaction (*F*(2, 3026) = 4.18, *p* = 0.02). Before the declaration, Hong Kong had the highest worry (*M* = 5.16), compared to the U.S. (*M* = 3.86, *p* < 0.001) and Singapore (*M* = 3.36, *p* < 0.001; the latter two, *p* < 0.001). After the declaration, worry increased in all three regions (Hong Kong: *M =* 5.41, *p* = 0.04, *d* = 0.17; U.S.: *M =* 4.62, *p* < 0.01, *d* = 0.40; Singapore: *M =* 3.69, *p* < 0.01, *d* = 0.20), but evidently, the increase was the most pronounced in the U.S.

#### Worry about not receiving treatment

Respondents indicated the extent to which they worried about not being able to receive treatment, personally, if they contracted COVID-19 (1 = *not at all*; 7 = *very much*; Table 1). The level of worry varied as a function of the declaration, region, and their interaction (*F*(2, 3026) = 3.13, *p* = 0.04). Before the declaration, Hong Kong had the highest worry (*M* = 4.96), compared to the U.S. (*M* = 3.66, *p* < 0.001) and Singapore (*M* = 3.36, *p* < 0.001; the latter two, *p* = 0.002). After the declaration, worry increased in all three regions (Hong Kong: *M =* 5.27, *p* = 0.02, *d* = 0.20; U.S.: *M =* 4.45, *p* < 0.01, *d* = 0.38; Singapore: *M =* 3.75, *p* < 0.01, *d* = 0.21), but evidently, the increase was the most pronounced in the U.S.

### Changes in behavioral responses

#### Preventative actions taken

We calculated the total number of preventative actions taken, out of the eight given, against the threat of COVID-19. This varied as a function of the declaration, region, and their interaction (*F*(2, 3026) = 3.04, *p* = 0.05). Before the declaration, people in Hong Kong were taking the most preventative actions (*M* = 5.52), compared to Singapore (*M* = 4.01) and the U.S. (*M* = 3.56); all pair-wise contrasts significantly different (*p*’s < 0.001). As might be expected, Hong Kong and Singapore were more behaviorally prepared for the outbreak than the U.S. was, possibly due to their expectations about the COVID-19 outbreak even before the pandemic declaration. (Compared to Singapore, Hong Kong had more widespread use of masks and practice of respiratory hygiene and social distancing, including mandatory school closure and work-from-home. These differences at the pre-declaration stage are not discussed here.) Of central relevance to this research is whether the declaration induced people to step up their preventative behaviors, or instead become paralyzed by fear. We found that after the pandemic declaration, the number of actions taken increased in both the U.S. (*M* = 3.86, *p* = 0.01, *d* = 0.20) and Singapore (*M* = 4.38, *p* < 0.01, *d* = 0.25). This number remained stable and high in Hong Kong (*M* = 5.55, *p* = 0.82, *d* = 0.02; Table 2).

**Table 2:** Actions and knowledge about precautionary measures before and after the WHO pandemic announcement, by region: Hong Kong, Singapore, and the U.S., 2020.

Variable	Hong Kong		Singapore		U.S.		Overall *F*-value (*p*)
	**Before**	**After**	**Before**	**After**	**Before**	**After**	
(*N* = 3,032)	763	257	697	308	834	173	
***Number of precautionary actions taken***							3.04 (0.05)
Mean	5.52	5.55	4.01	4.38	3.56	3.86	
*SD*	1.45	1.37	1.44	1.5	1.51	1.46	
95% CI for difference	(−0.18, 0.23)		(0.17, 0.57)		(0.07, 0.55)		
***Number of product categories stockpiling***							2.04 (0.13)
Mean	3.22	3.9	1.21	1.57	1.92	2.78	
*SD*	2.34	2.28	1.96	2.21	2.64	3.06	
95% CI for difference	(0.47, 1.25)		(0.34, 1.02)		(0.04, 0.68)		
***Number of WHO recommendations correctly identified***							0.06 (0.94)
Mean	2.63	2.64	2.89	2.92	2.61	2.61	
*SD*	0.75	0.75	0.79	0.85	0.82	0.82	
95% CI for difference	(−0.11, 0.12)		(−0.08, 0.13)		(−0.13, 0.13)		

SD = standard deviation; CI = confidence interval.

The proportions of respondents taking each action are summarized in Table 3. Here, we highlight the overall trend across regions (last two columns). Before versus after the declaration, 64.5% vs. 72.2% (χ^2^(1) = 15.02, *p* < 0.001) of respondents practiced social distancing; 76.9% vs. 80.9% (χ^2^(1) = 5.07, *p* = 0.02) avoided touching eyes, nose, and mouth; 41.9% vs. 49.2% (χ^2^(1) = 15.03, *p* < 0.001) wore a mask whenever going out; and 37.4% vs. 41.5% (χ^2^(1) = 3.98, *p* = 0.05) disinfected items they brought home. Moreover, 16% of respondents reported taking a deep-breath self-check test–a “fake cure” that has been debunked [21]–after the declaration, compared to the 12.6% before (χ^2^(1) = 5.37, *p* = 0.02).

**Table 3:** Precautionary actions breakdown before and after the WHO pandemic announcement, by region: Hong Kong, Singapore, and the U.S., 2020.

Variable	Hong Kong		Chi-square test (*p*)	Singapore		Chi-square test (*p*)	U.S.		Chi-square test (*p*)	Total		Chi-square test (*p*)
	Before	After		Before	After		Before	After		Before	After	
(*N* = 3,032)	763	257		697	308		834	173		2,294	738	
***Wash your hands frequently*** ^***a***^												
% taking action	95.8	97.7	1.85 (0.17)	95.3	95.1	0.01 (0.93)	92.4	91.3	0.25 (0.62)	94.4	95.1	0.54 (0.46)
% claiming as recommended (rcmd)	86.4	85.2	0.21 (0.64)	92.8	91.6	0.49 (0.48)	90.0	89.0	0.17 (0.68)	89.7	88.8	0.50 (0.48)
***Maintain social distancing*** ^***a***^												
% taking action	78.2	76.7	0.28 (0.60)	62.4	68.8	3.84 (0.05)	53.6	71.7	19.08 (< 0.001)	64.5	72.2	15.02 (< 0.001)
% claiming as rcmd	56.5	61.9	2.28 (0.13)	57.8	69.8	12.96 (< 0.001)	48.9	66.5	17.69 (< 0.001)	54.1	66.3	33.48 (< 0.001)
***Avoid touching eyes*, *nose and mouth*** ^***a***^												
% taking action	86.2	86	0.01 (0.92)	79.5	81.8	0.73 (0.39)	66.3	71.7	1.88 (0.17)	76.9	80.9	5.07 (0.02)
% claiming as rcmd	75.6	73.9	0.29 (0.59)	88.5	84.1	3.75 (0.05)	83.2	76.9	3.92 (0.05)	82.3	78.9	4.38 (0.04)
***Practice respiratory hygiene*** ^***a***^												
% taking action	78.5	77.8	0.05 (0.82)	45.3	47.4	0.37 (0.54)	32.7	31.2	0.15 (0.70)	51.8	54.2	1.30 (0.25)
% claiming as rcmd	45	42.8	0.36 (0.55)	49.6	46.1	1.07 (0.30)	38.4	28.3	6.23 (0.01)	44.0	40.8	2.33 (0.13)
***Do a deep-breath self-check every morning*** ^***b***^												
% taking action	11.5	14.0	1.10 (0.29)	14.1	17.2	1.66 (0.20)	12.5	16.8	2.30 (0.13)	12.6	16	5.37 (0.02)
% claiming as rcmd	12.3	11.3	0.19 (0.66)	18.5	19.5	0.13 (0.72)	24.2	23.7	0.02 (0.88)	18.5	17.6	0.31 (0.58)
***Drink water and keep your throat moist*** ^***b***^												
% taking action	55.7	51.8	1.21 (0.27)	61	67.2	3.56 (0.06)	53	49.1	0.86 (0.35)	56.3	57.6	0.37 (0.55)
% claiming as rcmd	19.7	18.3	0.23 (0.63)	41.3	38.6	0.62 (0.42)	39.2	42.2	0.53 (0.47)	33.3	32.4	0.23 (0.63)
***Wear a face mask whenever you go out*** ^***b***^												
% taking action	94.4	94.6	0.01 (0.91)	2.4	3.8	13.00 (< 0.001)	9.6	14.5	3.62 (0.06)	41.9	49.2	15.03 (< 0.001)
% claiming as rcmd	8.2	8.5	0.01 (0.91)	27.0	27.3	0.01 (0.92)	27.8	30.1	0.36 (0.55)	45.0	46.5	0.50 (0.48)
***Disinfect items you bring back home*** ^***b***^												
% taking action	52.0	56.4	1.49 (0.22)	23.5	29.9	4.52 (0.03)	35.5	39.9	1.20 (0.27)	37.4	41.5	3.98 (0.05)
% claiming as rcmd	24.4	26.1	0.30 (0.59)	24.4	23.1	0.21 (0.65)	48.2	43.4	1.35 (0.25)	33.0	28.9	4.48 (0.03)

^a^ Precautionary items recommended by the WHO.

^b^ Precautionary items recommended by social media sources.

#### Stockpiling

We listed ten product categories (e.g., toilet paper, hand sanitizer, bottled water) and asked respondents to indicate which they had stockpiled. We summed the number of categories in which they reported stockpiling. There was no interaction between region and pandemic declaration (ANOVA *F*(2, 3026) = 2.04, *p* = 0.13), but main effects of both region (*F*(2, 3026) = 169.59, *p* < 0.001) and declaration (*F*(1, 3026) = 37.71, *p* < 0.001). Before the declaration, Hong Kong stockpiled the most categories (*M* = 3.22), compared to the U.S. (*M* = 1.92, *p* < 0.001) and Singapore (*M* = 1.21, *p* < 0.001; the latter two, *p* < 0.001). After the declaration, stockpiling increased everywhere (Hong Kong: *M* = 3.90, *p* < 0.001, *d* = 0.29; U.S.: *M* = 2.78, *p* < 0.001, *d* = 0.30; Singapore: *M* = 1.57, *p* = 0.03, *d* = 0.17; Table 2).

#### Correlation with psychological responses

Our findings suggest that people stepped up (or kept up, as in Hong Kong) their preventative actions and stockpiled more after the pandemic declaration, rather than giving up on the fight against the disease. Were the people who stockpiled also the ones who were fearful, and stepped up their preventative actions? Individual-level data show, across regions, that those who took more precautionary actions tended to be more anxious (Hong Kong: *r* = 0.14, *p* < 0.01; Singapore: *r* = .15, *p* < 0.01; U.S.: *r* = 0.12, *p* < 0.01), more worried about the healthcare system’s capacity (Hong Kong: *r* = 0.13, *p* < 0.01; Singapore: *r* = 0.09, p < 0.01; U.S.: *r =* 0.14, *p* < 0.01), more worried about not receiving treatment personally (Hong Kong: *r* = 0.16, *p* < 0.01; Singapore: *r* = 0.09, *p* < 0.01; U.S.: *r* = 0.18, *p* < 0.01), and stockpiled more (Hong Kong: *r* = 0.27, *p* < 0.01; Singapore: *r* = 0.24, *p* < 0.01; U.S.: *r* = 0.34, *p* < 0.01; S4 Appendix in S1 File). Overall, these results indicate that while the pandemic announcement did increase anxiety, by and large, it had the intended effects of prompting people to step up their preventative actions.

## General discussion

We found significant changes in psychological and behavioral responses before and after the WHO’s declaration of COVID-19 as a pandemic, across Hong Kong, Singapore, and the U.S. Across regions, people were (reasonably) more concerned about the healthcare system’s capacity and their own ability to receive treatment after the pandemic declaration, with the effect most pronounced in the U.S. Moreover, people in the U.S. experienced greater increase in generalized anxiety post-declaration than those in Hong Kong and Singapore. There was a sharp increase (from 17.5% to 32.9%) in the proportion of people who crossed the clinical threshold for GAD in the U.S., but not in Singapore or Hong Kong. These findings are consistent with the notion that psychological reactions may be stronger among people in a region that has less experience of the outbreak and more relaxed official measures [35].

Respondents in the U.S. and Singapore, but not in Hong Kong, reported taking more actions after the pandemic declaration. One reason might be that people in Hong Kong had already taken various preventative measures since January, two months before the pandemic declaration [35]. Among the U.S. and Singapore, the increase in the adoption of social distancing was particularly remarkable. It is possible to attribute this to the declaration of national emergency on March 13^th^ in the U.S., and the closure of schools and stores afterwards, but there was no such declaration and schools remained open in Singapore during this period. Hence it is likely that the pandemic declaration influenced that increased adoption of social distancing. Relatedly, one may draw a connection between Singapore’s increased practice of social distancing and its relatively low generalized anxiety–evidently, a pandemic declaration may have positive effects on behavioral change without causing anxiety and fear. Less reassuringly, there was an overall increase in the adoption of a “fake cure”–the deep-breath self-check test–after the pandemic declaration. Thus, while increased community engagement was a positive outcome, some people fell prey to false information [36].

Our research contributes to the ongoing conversation about the social and institutional effects of declaring a pandemic. For example, Chan and colleagues’ analysis of Google mobility data showed that the WHO’s pandemic declaration significantly reduced mobility at the country level, and that the decline in mobility can be attributed to risk attitudes [37]. Jun, Yoo and Lee further examined Google search volumes and showed that the pandemic declaration increased searches for information on COVID by more than 20% globally [38]. Our findings make a unique contribution to this literature by linking psychological and behavioral responses at the individual level, and showing that there are immediately observable effects on laypeople’s psychological and behavioral responses. Some of these responses are positive (e.g., an increase in social distancing, and increases in reasonable concerns about the healthcare system’s capacity), and should contribute to “flattening the curve.” However, other responses (e.g., increases in generalized anxiety and stockpiling) are negative, and may have serious second-order effects on mental health and daily life. We observe both effects, and some occur simultaneously in the same region. Moreover, the patterns of responses vary across geographic regions, with the differences appearing consistent with social and political differences, and differences in the local onset and progression of the breakout. Indeed, these regions vary in multiple dimensions and demographic and geographic differences beyond the scope of this research may well also contribute to the observed regional variations. The case of Singapore, where people increased social distancing without becoming more anxious after the declaration, suggests that the word “pandemic” can possibly induce positive changes without causing fear.

### Implications

Our findings have important public health implications. The first implication relates to the “reasonableness” of the fear induced by the pandemic announcement. We find that the increase in fear is non-trivial, but possibly not unreasonable. People who were experiencing the onset of the disease during the period of data collection (i.e., U.S., an early stage country) manifested increased “reasonable” fear (i.e., fear about exceeding the healthcare system’s capacity, and about not receiving treatments) to a greater extent than people in regions that had experiences, and where control measures had taken effect (in Hong Kong and Singapore) in the respective period. Indeed, as the pandemic progresses and risk perceptions change, the public’s anxiety levels adjust accordingly [39]. It is also important to note that our data do not shed any light on the optimal timing of the pandemic declaration. Rather, they provide evidence that the declaration of a pandemic does have systematic effects on the general public. While our research is not a clinical screening for mental health issues, the observed increase in fear and anxiety in response to the declaration of a pandemic highlights the importance of interventions that safeguard mental wellbeing at the time of such a declaration.

The second implication relates to the role of expectations. The public had been exposed to news about COVID-19 well before the declaration of a pandemic. Therefore, responses would have been driven not just by the fear-inducing elements of the declaration, but also by expectations salient at the time. According to the expectancy confirmation theory, people’s responses to an announcement pivot critically on whether the announcement carries information that confirms or disconfirms their existing expectancy. Expectancy confirmation signals to oneself that no additional actions are required. In contrast, a disconfirmed expectancy calls for heightened vigilance and corrective actions [40]. This suggests that, in the case of the COVID-19 pandemic declaration, the increase of fear and actions would be lower if the public has a prior expectation about the disease outbreak and is prepared for the announcement. Indeed, we observe the increase of fear and actions being least pronounced in Hong Kong, compared to the U.S. or Singapore. This regional variation may be explained by different expectations of the outbreak in these regions, based on the stage of the outbreak and the local officials’ responses (e.g., Hong Kong was months into the outbreak and had had multiple preventative measures implemented when the declaration was made, whereas Singapore and the U.S. had more relaxed measures, and the U.S. was only at the early stage of outbreak). Taking expectancies into consideration, the WHO’s decision should be less about whether to declare a pandemic (driven by considerations of fear), but more about how to plan the staging and timing of the declaration to shape expectancies. Consequently, there should be a fundamental shift in future research and policy efforts–to proactively measure and manage public expectations of a disease outbreak as it evolves.

The third implication relates to the need for governments to better understand public psychology. The general public’s psychological and behavioral responses to any public health measures determine the success or failure of these measures’ implementation. Over the past two years, there is ample evidence from across the world that people vary in their responses to the same public health measure or communication as a function of individual and societal factors. Individuals’ perceptions and psychological reactions (e.g., fear and anxiety) toward COVID-19 determine their trust and compliance toward public health measures [41–43]. Thus, understanding the psychology of the public in crisis situations, such as a pandemic, is an important first step to devising effective public health strategies and communications.

### Concluding remarks

On March 10, 2022, two years after the declaration of COVID-19 as a pandemic, Dr. Mike Ryan, the Executive Director of the WHO Health Emergencies Program, offered a look-back at 2020 when the PHEIC was issued:

*We were trying to get people to act without panicking*. *Trying to get people to act without stumbling*. *Trying to get people to take action without generating a sense of panic amongst ordinary people*. *We were ringing the bell but people weren’t enacting*. *Our mistake was we listened to the wrong alarm*. *We waited until someone was ringing a bell on top of the last patch of dry land going “It’s a pandemic*.*” That doesn’t help anymore but what should have helped was the announcement at the end of January (2020)*. *That’s what should have triggered the global action*.*”* [44]

Is it pragmatic to hope for a massive scale of collective actions at the community level without triggering fear in the general public? Fear is a vital response to danger; it mobilizes actions and has played a critical protective role in human evolution. In public health, the lack of fear towards certain diseases is often associated with inaction (e.g., people are less motivated to combat noncommunicable diseases such as diabetes and obesity as they are not fearful of these diseases) [45]. While the attempt to not trigger fear is certainly appealing, the agenda to induce large-scale collective actions without inducing fear may have proved a touch idealistic [16]. Our findings, which suggest that the declaration of pandemic triggered a reasonable level of fear and an increased level of preventative actions, highlight the importance of signaling seemingly negative emotions (e.g., fear and anxiety) and understanding the importance of directing appropriately. As COVID-19 is unlikely to be the last pandemic we face, public health will benefit from a more holistic view of emotional regulations, which switch from seeing fear as an irrational and unpleasant emotion that should be avoided, to acknowledging it as an important component in the regulation of behaviors. This would, in turn, inform the decision rules that guide the development of quantitative models for policy decisions [46]. Looking back two and half years into the pandemic, as the virus has mutated and vaccines become available, national and regional public health officials have grown to play bigger roles thereby taking divergent directions in shaping public responses to the pandemic. These emergent directions should not obscure the importance of the WHO’s declaration at the beginning of the outbreak. When the virus was novel and the situation unclear, the WHO’s message was crucial in setting the stage for regional public health officials to implement preventative measures and engage community actions.

## Supporting information

S1 File (DOCX)
