# Supplementary material for: Psychological and behavioral responses to the declaration of COVID-19 as a pandemic: A comparative study of Hong Kong, Singapore, and the U.S
Source: PLoS One. 2022 Oct 10;17(10):e0275854. doi: 10.1371/journal.pone.0275854 (PMC9551632; doi:10.1371/journal.pone.0275854)
Supplement: S1 File — (DOCX) [file pone.0275854.s001.docx]

**S1 Appendix: Descriptive Summary of Sample by Region**

| **Variable** | **Hong Kong** | | **Singapore** | | **U.S.** | |
| --- | --- | --- | --- | --- | --- | --- |
|  | **Before, %** | **After,**  **%** | **Before, %** | **After,**  **%** | **Before, %** | **After,**  **%** |
| (*N* = 3,032) | 763 | 257 | 697 | 308 | 834 | 173 |
| **Gender** |  |  |  |  |  |  |
| Male | 47.6 | 38.9 | 51.6 | 43.5 | 46.9 | 65.3 |
| Female | 52.4 | 61.1 | 48.4 | 56.5 | 53.1 | 34.7 |
| **Age** | | | | | | |
| < 18 | 1.4 | 8.2 | 1.0 | 0.6 | 1.6 | 1.2 |
| 18–24 | 13.9 | 16.3 | 17.9 | 9.7 | 10.4 | 16.8 |
| 25–34 | 19.7 | 17.9 | 14.3 | 28.9 | 15.8 | 21.4 |
| 35–44 | 18.7 | 21.0 | 15.1 | 27.6 | 16.9 | 16.2 |
| 45–54 | 19.4 | 19.8 | 17.4 | 21.4 | 17.9 | 19.7 |
| 55–64 | 21.5 | 9.7 | 22.5 | 5.2 | 18.0 | 11.0 |
| ≥ 65 | 5.4 | 7.0 | 11.8 | 6.5 | 19.4 | 13.9 |
| **Education level** | | | | | | |
| Primary school | 0.9 | 2.7 | 0.4 | 0.6 | 0.6 | 1.2 |
| Middle school | 4.5 | 3.5 | 11.6 | 7.1 | 1.3 | 0.6 |
| High school | 32.0 | 35.0 | 38.0 | 29.2 | 36.7 | 45.7 |
| University or above | 62.6 | 58.8 | 49.9 | 63.0 | 61.4 | 52.6 |
| **Annual personal income, $** | | | | | | |
| < 50,000 | 12.8 | 19.5 | 34.6 | 26.9 | 14.4 | 8.7 |
| 50,000–99,999 | 4.1 | 5.1 | 11.6 | 10.7 | 12.9 | 11.0 |
| 100,000–199,999 | 15.2 | 10.1 | 9.5 | 15.9 | 13.4 | 9.8 |
| 200,000–299,999 | 18.9 | 15.6 | 8.9 | 7.1 | 10.1 | 14.5 |
| 300,000–399,999 | 17.6 | 14.8 | 7.0 | 9.7 | 8.6 | 11.0 |
| 400,000–499,999 | 9.0 | 12.8 | 5.7 | 8.4 | 8.0 | 9.2 |
| 500,000–599,999 | 6.2 | 6.6 | 4.6 | 5.5 | 6.4 | 7.5 |
| 600,000–699,999 | 5.6 | 6.6 | 3.3 | 4.5 | 7.0 | 6.9 |
| 700,000–799,999 | 3.4 | 3.1 | 4.3 | 2.3 | 4.1 | 1.7 |
| 800,000–899,999 | 2.6 | 3.9 | 6.6 | 6.2 | 3.7 | 3.5 |
| 900,000–999,999 | 1.3 | 1.2 | 2.0 | 1.9 | 6.7 | 8.7 |
| ≥ 1,000,000 | 3.3 | 0.8 | 1.9 | 0.6 | 4.7 | 7.5 |

**S2 Appendix: Analyses on Source Manipulation**

After respondents indicated the preventative actions they had been taking, personally, we indicated to the respondents either the four actions that were recommended by the WHO (i.e., the WHO condition), or the four actions sourced from social media (i.e., the social media condition). This manipulation had no effect on the psychological and behavioral variables that were measured after the administration of the manipulation and were examined in this paper. However, it affected respondents’ trust in various sources, which are not the focus on this paper and, hence, not reported or discussed in the paper. Results of the analyses on this manipulation are summarized below:

| DVs | *M*_social-media-condition_ | *M*_WHO-condition_ | *Statistics* |
| --- | --- | --- | --- |
| Generalized anxiety | 5.27 | 5.41 | *F*(1, 3030) = 0.52, *p* = 0.47 |
| Worry about healthcare capacity | 4.23 | 4.22 | *F*(1, 3030) = 0.09, *p* = 0.76 |
| Worry about not receiving treatment | 4.11 | 4.11 | *F*(1, 3030) < 0.001, *p* = 0.99 |
| Stockpiling | 2.24 | 2.29 | *F*(1, 3030) = 0.03, *p* = 0.58 |
| Trust in the WHO | 4.30 | 4.51 | *F*(1, 3030) = 9.74, *p* = 0.002 |
| Trust in the government | 4.23 | 4.32 | *F*(1, 3030) = 1.33, *p* = 0.25 |
| Trust in other residents | 4.47 | 4.47 | *F*(1, 3030) < 0.001, *p* = 0.99 |
| Trust in social media | 4.01 | 4.15 | *F*(1, 3030) = 5.25, *p* = 0.02 |

**S3 Appendix: Key Measures in Survey**

***I. Preventative Actions Correctly Identified*** (results not discussed but summarized in Table 2)

What precautions should a normal healthy adult take to protect themselves from COVID-19? We have gathered 8 recommendations from different sources. These recommendations are shown below. Please read them carefully and indicate the FOUR that medical professionals recommend as the most important:

1. Wash your hands frequently

Regularly and thoroughly clean your hands with an alcohol-based hand rub or wash them with soap and water.

Why? Washing your hands with soap and water or using alcohol-based hand rub kills viruses that may be on your hands.

2. Maintain social distancing

Maintain at least 1 meter (3 feet) distance between yourself and anyone who is coughing or sneezing.

Why? When someone coughs or sneezes they spray small liquid droplets from their nose or mouth which may contain the virus. If you are too close, you can breathe in the droplets, including the COVID-19 virus if the person coughing has the disease.

3. Avoid touching eyes, nose and mouth.
Why? Hands touch many surfaces and can pick up viruses. Once contaminated, hands can transfer the virus to your eyes, nose or mouth. From there, the virus can enter your body and can make you sick.

4. Practice respiratory hygiene

Make sure you and the people around you follow good respiratory hygiene. People should cover their mouths and noses with their bent elbows or tissue when they cough or sneeze. Then dispose of the used tissue immediately.

Why? Droplets spread virus. By following good respiratory hygiene, people can be protected from viruses such as cold, flu and COVID-19.

5. Do a deep-breath self-check every morning to achieve early diagnosis of infection.

Why? The new coronavirus may not show sign of infection for many days, so people may not get diagnosed early. By the time they have fever and/or cough and go to the hospital, the lungs may have up to 50 % fibrosis and it is too late!
Taiwanese experts provide a simple self-check that we can do every morning: Take a deep breath and hold your breath for more than 10 seconds. If you complete it successfully without coughing, without discomfort, stuffiness or tightness, it proves there is no fibrosis in the lungs and indicates no infection.

6. Drink water and keep your throat moist.

Why? Japanese doctors treating COVID-19 cases advise that everyone should ensure your mouth & throat are moist, never dry. Take a few sips of lukewarm water every 15 minutes at least. Even if the virus gets into your mouth, the virus will be washed down through your esophagus and into the stomach when you drink water. Your stomach acid will kill all the virus. If you do not drink water regularly, the virus can enter your windpipes and into the lungs.

7. Wear a face mask whenever you go out.

Why? Most people touch their nose/mouth more than 90 times a day without knowing it. By wearing a face mask, you will stop touching your nose and mouth mindlessly.

8. Disinfect items you bring back home, including your phone, computer, jackets, and everything you buy from outside. Either spray alcohol on them or wipe with diluted bleach.
Why? Contact transmission happens when an individual touches an object contaminated with droplets containing virus. The virus can then spread if the person touches his nose, mouth, or eyes. COVID-19 can survive on materials such as plastic bags, plastic containers, phones, and other surfaces for hours or even days. To reduce the chance of you and your family getting infected at home due to contact transmission, you should disinfect items that are brought back home.

Now Please indicate the FOUR that medical professionals worldwide recommend as the most important:

Wash your hands frequently

Maintain social distancing

Avoid touching eyes, nose and mouth

Practice respiratory hygiene

Do a deep-breath self-check every morning to achieve early diagnosis of infection.

Drink water and keep your throat moist.

Wear a face mask whenever you go out.

Disinfect items you bring back home, including your phone, computer, jackets, and everything you buy from outside. Either spray alcohol on them or wipe with diluted bleach.

***II. Preventative Actions Taken***

Now consider the same list of precautions again. Are you taking any of these precautions? Please indicate the ones you are regularly taking. You can choose as many as applicable.

Wash your hands frequently

Maintain social distancing

Avoid touching eyes, nose and mouth

Practice respiratory hygiene

Do a deep-breath self-check every morning to achieve early diagnosis of infection.

Drink water and keep your throat moist.

Wear a face mask whenever you go out.

Disinfect items you bring back home, including your phone, computer, jackets, and everything you buy from outside. Either spray alcohol on them or wipe with diluted bleach.

***III. Source Manipulation*** (not examined in the current paper; see S2 Appendix)

*(The WHO condition read the following)*

As we mentioned earlier, the 8 recommendations are gathered from different sources. The 4 listed below are provided by the WHO (World Health Organization) and are recommended by medial professionals worldwide.

Wash your hands frequently

Maintain social distancing

Avoid touching eyes, nose and mouth

Practice respiratory hygiene

*(The social media condition read the following)*

As we mentioned earlier, the 8 recommendations are gathered from different sources. The 4 listed below are gathered from social media sources, and are widely shared by many people.

Do a deep-breath self-check every morning to achieve early diagnosis of infection.

Drink water and keep your throat moist.

Wear a face mask whenever you go out.

Disinfect items you bring back home, including your phone, computer, jackets, and everything you buy from outside. Either spray alcohol on them or wipe with diluted bleach.

***IV: Generalized anxiety***

Over the last 2 weeks, how often have you been bothered by the following problems?

Feeling nervous, anxious, or on edge

Not being able to stop or control worrying

Worrying too much about different things

Trouble relaxing

Being so restless that it’s hard to sit still

Becoming easily annoyed or irritated

Feeling afraid as if something awful might happen

(0 = *not at all sure*, 1 = *several days*, 2 = *over half the days*, 3 = *nearly every day*)

***V. Worries about healthcare capacity and not receiving treatment***

No one knows how exactly how long the COVID-19 outbreak will last and how it will evolve overall, but we want to know your speculation of what may happen.

1. What percent of the population in [region] may eventually contract COVID-19 by the end of the outbreak? _____ (enter a number between 0–100)
2. How likely is it that the outbreak of COVID-19 in [region] will go beyond the healthcare system’s capacity? (1 = *very unlikely*; 7 = *very likely*)
3. How worried are you that you may not be able to receive appropriate treatment if you contract COVID-19, because your local healthcare system may be overwhelmed by the outbreak? (1 = *not worried at all*; 7 = *very worried*)

[Item 1 was not examined in the paper because a significant proportion of participants in all three regions reported responses less than 1 (e.g., 0.3). It is unclear whether such a response indicates 0.3% or 30%. Hence this measure might be unreliable due to confusion in the wording of the question. Item 2 is the measure for worry about healthcare capacity. Item 3 is the measure for worry about not receiving treatment.]

***VI. Stockpiling***

Have you stockpiled the following items for COVID-19?

Staple foods such as pasta, rice, flour ____

Toilet paper ___

Hand sanitizers ___

Face masks ___

Alcohol wipe ___

Canned food ___

Water ____

Vitamins ___

Soda and soft drinks ____

Prescription medications ___

None of the above

***VII. Trust in various sources*** (not examined in the current paper; see S2 Appendix)

Please indicate the extent to which you agree with each of the following statements in the context of the Covid-19 coronavirus epidemic: (1 = *not at all*; 7 = *very much*)

I trust the WHO to make recommendations that serve the best interests of all regions of the world.

I trust the government of [region] to make recommendations that serve the best interests of people in [region].

I trust that the residents of [region] will act in ways that are in the best interests of [region].

I trust that social media will communicate information that is in the best interests of [region].

**S4 Appendix: Correlational Results by Region**

| **Variable** | **Hong Kong** | **Singapore** | **The U.S.** |
| --- | --- | --- | --- |
| *N* | 1,020 | 1,005 | 1,007 |
| **Correlation between *knowledge about WHO recommendations* and the following variables** | | | |
| Total preventative actions taken |  | | |
| Pearson's *r* | −0.05 | −0.06 | 0.02 |
| *p* | 0.15 | 0.05 | 0.58 |
| Stockpiling |  |  |  |
| Pearson's *r* | −0.06 | −0.16 | −0.12 |
| *p* | 0.06 | < 0.001 | < 0.001 |
| Generalized anxiety |  |  |  |
| Pearson's *r* | −0.04 | −0.15 | −0.06 |
| *p* | 0.19 | < 0.001 | 0.07 |
| Worry that the outbreak will exceed the healthcare system's capacity | | |  |
| Pearson's *r* | 0.001 | −0.10 | −0.02 |
| *p* | 0.96 | < 0.001 | 0.52 |
| Worry about not being able to receive treatment personally | | | |
| Pearson's *r* | 0.004 | −0.09 | −0.05 |
| *p* | 0.88 | < 0.001 | 0.11 |
| **Correlation between *total number of preventative actions taken* and the following variables** | | | |
| Stockpiling |  | | |
| Pearson's *r* | 0.27 | 0.24 | 0.34 |
| *p* | < 0.001 | < 0.001 | < 0.001 |
| Generalized anxiety |  |  |  |
| Pearson's *r* | 0.14 | 0.15 | 0.12 |
| *p* | < 0.001 | < 0.001 | < 0.001 |
| Worry that the outbreak will exceed the healthcare system's capacity | | |  |
| Pearson's *r* | 0.13 | 0.09 | 0.14 |
| *p* | < 0.001 | < 0.01 | < 0.001 |
| Worry about not being able to receive treatment personally | | |  |
| Pearson's *r* | 0.16 | 0.09 | 0.18 |
| *p* | < 0.001 | < 0.01 | < 0.001 |
| **Correlation between *stockpiling* and the following variables** | | | |
| Generalized anxiety |  | | |
| Pearson's *r* | 0.27 | 0.26 | 0.20 |
| *p* | < 0.001 | < 0.001 | < 0.001 |
| Worry that the outbreak will exceed the healthcare system's capacity | | |  |
| Pearson's *r* | 0.22 | 0.23 | 0.21 |
| *p* | < 0.001 | < 0.001 | < 0.001 |
| Worry about not being able to receive treatment personally | | |  |
| Pearson's *r* | 0.29 | 0.20 | 0.31 |
| *p* | < 0.001 | < 0.001 | < 0.001 |
